# 

**Published:** 1893-11

**Authors:** 


### LITERARY.

A New Illustrated Dictionary of Medicine, Biology, and Collateral Sciences. P. Blakiston, Son & Co., Philadelphia, Publishers.

Dr. George M. Gould, already well known as the editor of two small Medical Dictionaries, has now about ready an unabridged, exhaustive work of the same class, upon which he and a corps of able assistants have been uninterruptedly engaged for several years.

The feature that will attract immediate attention is the large number of fine illustrations that have been included, many of which—as for instance, the series of over fifty of the bacteria—have been drawn and engraved especially for the work. Every scientific-minded physician will also be glad to have defined several thousand commonly used terms in biology, chemistry, etc. The chief point, however, upon which the editor relies for the success of his book is the unique epitomization of old and new knowledge. It contains a far larger number of words than any other one volume medical lexicon. It is a new book, not a revision of the older volume. The pronunciation etymology, definition, illustration, and logical groupings of each word are given. There has never been such a gathering of new words from the living literalure of the day. It is especially rich in tabular matter, a method of presentation that focuses, as it were, a whole subject so as to be understood at a glance.

The latest method of spelling certain terms, as adopted by various scientific bodies and authorities, have all been included, as well as those words classed as obsolete by some editors, but still used largely in the literature of to-day, and the omission of which in any work aiming to be complete would make it unreliable as an exhaustive work of reference.

The publishers announce that, notwithstanding the large outlay necessary to its production on such an elaborate ylan, the price will be no higher than that of the usual medical text-book.

The ATLANTIC MONTHLY for November carries on two serials, Mrs. Cavazza's "The Man from Aidone," and Charles Egbert Craddock's "His Vanished Star," and contains the second paper of Mr. W. F. Apthorp's "Two Modern Classicists in Music." This deals with Otto Dressel, a musician far less widely known than he deserved to be. Immediately following this article, which necessarily insists somewhat upon musical "schools," comes Mr. Owen Wister's paper on "Catholicity in Musical taste," a strong plea for the equal enjoyment of all sorts of good music. Amateurs of music will care especially for these two papers, well timed for the opening of the musical season. Mr. Bradford Torrey, in "Along the Hillsborough" brings to his readers another bit of the bird-lover's Florida. Outdoor and indoor England appear in Miss Alice Brown's "A Pilgrim in Devon," and Sir Edward Strachey's charming "Talk at a Country House," on Books, Tennyson and Maurice. In "Courts of Conciliation in America," Mr. Nicolay Grevstad tells of the grafting of a Scandinavian method of arbitration upon the laws of our own Northwest,—a most significant movement. From Wisconsin, through Mr. H. E. Scudder's "School Libraries," comes a clear showing of what the state can do in the cause of good reading. Ma. Ernest Hart, on "Spectacled Schoolboys," provides the remaining educational paper of the number, unless Miss Emily James Smith's "Hungary Greeklings," an entertaining study of decadent Greek teachers in Rome, be counted in the same class. "The Beautiful Loup-Garou," is a short story by Mrs. Catherwood, and the poems are "Morn after Morn," by Stewart Sterne, and "An Ionian Frieze," by Francis Howard Williams. At the end, as usual, come reviews and the Contributors' Club, Houghton.—Mifflin & Co., Boston.

A magazine is usually satisfied with one strong feature for the month. THE COSMOPOLITAN, however, presents for November, no less than five very unusual ones. William Dean Howells' gives the first of the letters of the traveller, who has been visiting this country from Altruria. We have read Mr. Howells' impression of the Altrurian ; but in this first letter we have the Altrurian's impressions of New York, with some comments upon our government and society, calculated to awaken the most conservative minds. The second feature of THE COSMOPOLITAN is the portion of the magazine given up to color work, no less than ten superb color illustrations being presented for the first time in magazine history, accompanying an article by Mrs. Roger A. Pryor on "Changes in Women's Costumes." The third feature is "American Notes," by Walter Besant, who was recently in America and is doing the United States for THE COSMOPOLITAN à la Dickens. The fourth feature is an article by General Badeau, on "The Forms of Invitation Used by the English Nobility." The article is illustrated by the facsimile of cards to the Queen's drawing-room, to dinner at the Princess of Wales, and to many leading houses of England. Finally we have a new and very curious story by Mark Twain, called "The Esquimau Maiden's Romance." It is in his happiest vein and is illustrated by Dan Beard. The November number presents the work of many artists, among whom are: C. S. Reinhart, Otto Guillonnet, J. H. Harper, G. Hudson, Franz von Lenbach, George Wharton, Edward F. Schuyler Matthews, Dan Beard, W. L. Sontag, Jr., F. G. Atwood, C. Hirschberg J. Habert-Dys, August Franzen, Louis J. Read, J. N. Hutchins and Hamilton Gibson.

McCLURE'S MAGAZINE, for November, opens with a dialogue between Frank R. Stockton and Edith M. Thomas. It is written in one of Miss Thomas's happiest moods, and is as bright as one of Sydney Smith's epigrams. Miss Thomas visits Mr. Stockton at his picturesque home, beyond the foothills of New Jersey, and there, under his own vine and fig tree, introduces us to him. And then we have the rare privilege of listening to two famous literary personages exchanging confidences, and telling how their poems and stories are conceived and written. It is a beautifully illustrated article, and No. 3 in the popular series of REAL CONVERSATIONS. There is, by the way, an excellent portrait of Mr. Stockton, which serves as frontispiece to this number.

INCURABLE, A GHETTO TRAGEDY, by that witty, thoughtful London author, I. Zangwill, is one of the most pathetic of short stories. Once begun, this story will be read to the bitter end.

THE HUMAN DOCUMENTS and BIOGRAPHICAL NOTES in this number of McCLURE'S MAGAZINE, every reader will look for and linger over. Conan Doyle is pictured here, as boy and man, five times ; here we see R. E Peary, C. E., U. S. N., the Arctic explorer, at five different ages ; Camille Flammarion, the French astronomer and writer, at three interesting periods of his meteoric career; and F. Hopkinson Smith at the age of seventeen, twenty-five, forty-five and to-day. E. J. Edwards writes on President Cleveland. The whole number is a good one.

The Century Co. has bought well nigh the complete literary "out-put" of Mark Twain during his year of residence abroad, and both *The Century* and *St.*

*Nicholas* will have serial stories by this popular humorist among the attractions of the new year. For *The Century* he has written a novel which is said to abound with humorous and dramatic incident, and in some chapters to be a revelation of tragic power. Its plot includes a most ingenious employment of science in the detection of crime. It is called "Pudd'n'head Wilson," and like "Huckleberry Finn," and "Tom Sawyer," is a story of a Mississippi steamboat town.

For the boy and girl readers of *St. Nicholas*, he has written "Tom Saw[illegible]r Abroad," being the adventures of Tom Sawyer, accompanied by Huckleberry Finn and the negro "Jim," in the Eastern hemisphere,—which is not reached in the ordinary way, but accidentally, as it were, and in a flying-machine.

Godey's Magazine for December, ready November, 15th, promises to be a most attractive number. Theodora B. Wilson—a writer not unknown to novel readers—has the complete story. It is called "Love Conquers," and is vigorous, healthy, and strong. The illustratious are by Florence K. Upton. Rose Coghlan writes about Personal Requisites of the Stage, an article which all who are interested in the modern drama should read; that popular and graceful writer, Margaret Lemon, tells about "A Flying Trip to Florida;" Forence Hull has a charming short story called "Apple Blossoms;" Olive Thorne Miller writes about "The Dove's Doings," and the poems are by Frank Demster Sherman, Ednah Proctor Clarke, Edward W. Barnard, and others. All the departments are up to their standards and the two exquisite water-color portraits are of Mrs. John Bloodgood, Jr., and Miss Angelica Schuyler Crosby, of New York.

We have received a booklet on Exercise for Pulmonary Invalids, by Charles Denison, A.M., M.D., of Denver, Col., and it is truly a practical little work.

Good Housekeeping, for November, is naturally a Thanksgiving number, and the spirit of the New England anniversary, now adopted throughout the country, pervades a large portion of the contents. Miss Maria Parloa contributes the leading paper, which is devoted to the great national festival; while the serial, "A Noble Girlhood," which has been running during the year, appropriately presents and occurrences with a Thanksgiving flavor. But there is also an abundance of practical matter, relating to all the interests of the household, and this standard magazine was never more valuable or more deservedly popular than now. It promises numerous features of special interest and value for the coming year. Clark W. Bryan Company, Springfield, Mass.

We take great pleasure in announcing to our readers the early publication of a work interesting and valuable to all, The Parliament of Religions at the Columbian Exposition. Will be issued complete in one large octavo volume, and will be a careful compilation of all of the proceedings—at once a fascinating story and a book of universal value. A narrative of the grandest achievement in modern religious history. The book contains origin of the Parliament of Re ligions, proceedings of every meeting of the Parliament, speeches delivered and papers read at every session of the noted gathering, the beliefs of the various religious denominations; opinions of eminent divines in regard to the Parliament; influence of the Parliament upon the religious thought of the world. Published by F. T. Neely, Chicago. 800 pages. Price: Cloth, $2.50; full sheep, $4.00.

Taken as a whole, THE BOOK OF THE FAIR, by Hubert Howe Bancroft, is probably the best presentation of the Columbian Exposition, historical and descriptive, which has been attempted. The plan is comprehensive, and yet not too extended, the aim being to give the Exposition entire in pictures and print, in one thousand imperial folio pages, that is to say twenty-five parts of forty pagss each. By taking the leading exhibits of a class as representative of the whole, giving the rest minor mention, to a greater or less extent, the entire round can be made, and yet the total result be a work not too cumbersome or expensive for the general public to handle and purchase.

The conversation of our oyster supply by Mr. Robert F. Walsh, opens the November POPULAR SCIENCE MONTHLY, and is followed by an admirable lecture on evolution and ethics, by Prof. Huxley, of the Sheldonian Theatre, Oxford. In this number appears the concluding half of the Magazine's account of Electricity at the World's Fair. A thoughtful essay appears on the Scientific Method with Children, by Henry L. Clapp.

The periodical always abounds with rich contributions.

A medical directory of the State of Connecticut has been issued by the Danbury Medical Printing Company, of Danbury, Conn. It contains a list of all the medical practitioners in the state, the various medical societies, all the dentists and dental societies, druggists and pharmaceutical societies, nurses and training schools for nurses, hospitals, &c. Price $1.00, delivered free by post.

One very excellent feature of the REVIEW OF REVIEWS is the condensed form in which the chapter under the caption "The Progress of the World," is tersely and briefly written.

These timely paragraphs add an important characteristic to the make-up of this excellent magazine. The November number is enriched by an excellent article by S. G. Thompson, entitled "Possibilities of the Great Northwest."

"The Future of Silver Production," is admirably treated by E. Benjamin Andrews. The number is replete.

The BLUE AND GRAY, for October, comes to our table with a new and attractive cover. The number is an interesting one throughout. General Horatio C. King writes on "My Recollections of War Times." "The Union and Confederate Veterans," is treated by Henry Clay Fairman.

Every old soldier should become a subscriber to this thorough patriotic publication.

LIPPINCOTT'S, for November, contains another complete novel by Mrs. Hungerford, entitled "An Unsatisfactory Lover."

No. 9, of LIPPINCOTT'S notable stories, entitled "The Rustlers," adds very much to the number. J. Armory Knox writes upon "How the Light Came," and it is an enjoyable contribution.

